# Brain organoid models of Huntington's disease shift the focus towards neurodevelopment

**DOI:** 10.1242/dmm.052510

**Published:** 2025-10-28

**Authors:** Wenqing Xu, Alessandro Prigione

**Affiliations:** ^1^ Faculty of Mathematics and Natural Sciences, Heinrich Heine University, 40225 Düsseldorf, Germany; ^2^ Department of General Pediatrics, Neonatology and Pediatric Cardiology, Medical Faculty, University Hospital Düsseldorf, Heinrich Heine University, 40225 Düsseldorf, Germany

## Abstract

Huntington's disease (HD) is traditionally viewed as an age-related disorder. Emerging evidence suggests that mutant huntingtin (mHTT) disrupts early neurodevelopment, although the contribution of developmental alterations to the late disease onset remains to be clarified. Leveraging human pluripotent stem cell-derived brain organoids, we and others are exploring how mHTT affects the developing human brain. These models reveal impaired neural progenitor organization and function, accompanied by a mitochondrial stress response, indicating reduced capacity to manage cellular stress. Enhancing mitochondrial health and promoting neural cell resilience may thus represent potential strategies for improving the brain's compensatory mechanisms, thereby prolonging a healthy state. These insights highlight a potential window of opportunity for therapeutic interventions. Targeting mitochondrial fitness and neurodevelopmental pathways at early stages – long before clinical symptoms emerge – could help prevent or delay disease onset and progression in affected individuals.

## Teaser

**Summary:** Increasing evidence indicates neurodevelopmental aspects in Huntington's disease. Early interventions promoting healthy brain development and mitochondrial fitness might lead to prevention or delay of the disease pathology.

## The traditional view of pathogenesis in Huntington's disease

Huntington's disease (HD) is a devastating neurodegenerative disorder caused by abnormal CAG trinucleotide repeat expansion in the gene huntingtin (*HTT*) (Bates et al., 2015; The Huntington's Disease Collaborative Research Group, 1993). Humans typically have ten to 35 CAG repeats; in HD, this number can increase to 36 or more (Nance et al., 1998). The disease exhibits an autosomal dominant pattern of inheritance (Ghosh and Tabrizi, 2018) and typically presents in adulthood with progressive basal ganglia atrophy, leading to involuntary jerky movements (chorea) together with cognitive and psychiatric symptoms (Aylward et al., 1997; Ross and Tabrizi, 2011). Neuronal loss appears first to affect striatal projection neurons (SPNs) (Reiner et al., 1988), which are crucial for motor control (Albin et al., 1989), followed by cortical neurons (Cudkowicz and Kowall, 1990).

The prevalent view of the disease mechanisms is that mutant HTT (mHTT) can acquire a toxic gain of function, leading to abnormal protein folding and aggregation (Arrasate and Finkbeiner, 2012; Duyao et al., 1995; Rubinsztein and Carmichael, 2003; Walker, 2007). This interpretation, supported by evidence of pathogenetic improvement following HTT reduction in HD animal models (Yamamoto et al., 2000), led to the development of treatments based on approaches to lower mHTT. However, these strategies have yet to demonstrate functional validity in clinical settings. The GENERATION HD1 trial (NCT03761849) was halted owing to detrimental effects (McColgan et al., 2023), but other trials are still ongoing (e.g. NCT05686551, NCT06254482, NCT06826612, NCT05032196, NCT05822908, NCT06585449, NCT06024265, NCT06873334). Remarkably, the trial NCT05243017 recently reported significantly slower disease progression with HTT-lowering gene therapy (Dolgin, 2025), although the detailed clinical outcomes still need to published. At the same time, given the unclear pathogenicity of mHTT aggregates (Arrasate and Finkbeiner, 2012; Arrasate et al., 2004; Hickman et al., 2022; Kuemmerle et al., 1999; Saudou et al., 1998; Slow et al., 2005), alternative mechanistic hypotheses should be put forward to explain HD pathogenesis.

Recent findings suggest that the instability of the CAG repeat region can cause progressive length increases in particular brain regions (Kacher et al., 2021; Kennedy and Shelbourne, 2000; Swami et al., 2009), leading to selective neuronal death (Handsaker et al., 2025). This age-dependent somatic CAG expansion can take place decades before clinical diagnosis (Scahill et al., 2025) and particularly affects cortico-striatal projections (Pressl et al., 2024). Oxidative damage exacerbates somatic CAG expansion (Jonson et al., 2013; Kovtun et al., 2007), possibly through genome instability and alterations in mismatch-repair genes (Chang et al., 2002; Iyer and Pluciennik, 2021), which can then drive the HD pathogenesis (Wang et al., 2025). Hence, perhaps mHTT is not toxic per se, but can become toxic in certain cell types under certain conditions.

… findings in animal models and in pre-symptomatic HD cases suggest that the neuronal pathology could start early during development

## The impact of HD on neurodevelopment

Accumulating evidence demonstrates that wild-type HTT plays a physiological role during brain development (Barnat et al., 2017; Bhide et al., 1996; Reiner et al., 2003). HTT function supports axonal transport (Vitet et al., 2020), axonal growth cone formation (Capizzi et al., 2022), synapse development (McKinstry et al., 2014), neural rosette formation (Sardo et al., 2012), neuronal migration (Tong et al., 2011) and overall neurogenesis (Godin et al., 2010).

The developmental role of HTT is highlighted by the fact that its depletion is embryonic lethal (Nasir et al., 1995; Zeitlin et al., 1995), and its selective inactivation in the brain causes progressive neurodegeneration in mice (Dragatsis et al., 2000). SPNs appear to be particularly sensitive to HTT depletion (Burrus et al., 2020). The presence of HTT might help decrease the cellular toxicity of mHTT (Leavitt et al., 2001) and could protect against excitotoxicity (Leavitt et al., 2006). Transient reduction of HTT during neurodevelopment is sufficient to cause HD-like phenotypes at later stages in mice (Arteaga-Bracho et al., 2016). Therefore, early loss of HTT function could contribute to HD pathogenesis.

Pathological mHTT can also be especially harmful during brain development. mHTT affects progenitor proliferation (Molina-Calavita et al., 2014), as well as migration, axonal growth and maturation of cortical neurons (Barnat et al., 2017; Blockx et al., 2012; Capizzi et al., 2022; McKinstry et al., 2014). During development, mHTT transiently alters the axonal projections and synaptic transmission of SPNs (Lebouc et al., 2025). This early vulnerability might be counteracted by compensatory mechanisms that could become insufficient over time. Indeed, HD-like features are recapitulated in mice by exposure to mHTT selectively during development (Molero et al., 2016).

Clinical studies in individuals with HD have uncovered pre-symptomatic alterations, including reduced growth (Lee et al., 2012), regional brain atrophy (Aylward et al., 2013; Scahill et al., 2013), impairment of synaptic function and cytoskeletal integrity (DiProspero et al., 2004), and defects in executive function (Pfalzer et al., 2023; Tabrizi et al., 2009). The cerebrospinal fluid of pre-symptomatic cases can contain elevated levels of neurofilament light protein, indicative of neuroaxonal damage (Scahill et al., 2025).

Neurodevelopmental abnormalities have also been observed in HD cases (van der Plas et al., 2019). These include developmental malformations, such as heterotopia, resulting from abnormal neuronal migration to the cortex (Hickman et al., 2021; Kubera et al., 2019; van der Plas et al., 2019), abnormal cortical surface (Mangin et al., 2020) and reduction of intracranial volume (Kubera et al., 2019; Mangin et al., 2020; Nopoulos et al., 2011; van der Plas et al., 2019). Cortical cell depletion correlates with transcriptional neurodevelopmental changes (Estevez-Fraga et al., 2023; Scahill et al., 2013). Remarkably, human fetuses carrying mHTT exhibit abnormalities in the developing cortex, with impaired proliferation of neural progenitors and premature differentiation (Barnat et al., 2020).

**Fig. 1.**
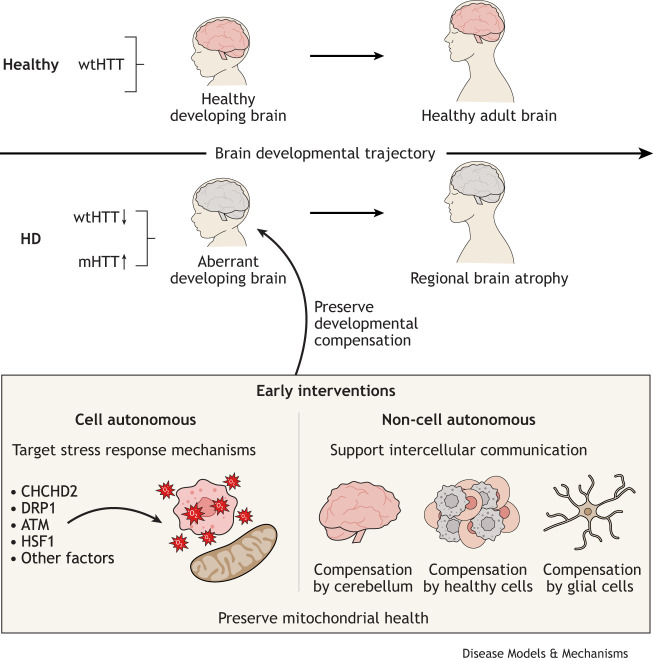
**Harnessing altered neurodevelopment in Huntington's disease for early treatment strategies.** Evidence from patients, animal models and human stem cell models demonstrate aberrant brain development occurring in Huntington's disease (HD). These demonstrations could help uncover alternative therapeutic avenues to promote brain resilience, aiming to maintain the compensatory mechanisms of the developing human brain as much as possible. Among these mechanisms, we highlight cell-autonomous approaches, for example based on modulating stress response mechanisms, and non-cell-autonomous approaches that could target inter-cell communication. Collectively, we propose the preservation of mitochondrial function as a central mechanism in early interventions for HD. ATM, ataxia telangiectasia mutated; CHCHD2, coiled-coil-helix-coiled-coil-helix domain containing 2; DRP1, dynamin-1-like protein; HD, Huntington's disease; HSF1, heat shock transcription factor 1; mHTT, mutant huntingtin; wtHTT, wild-type huntingtin.

Collectively, findings in animal models and in pre-symptomatic HD cases suggest that the neuronal pathology could start early during development (Table 1). Loss of wild-type HTT coupled with the presence of pathogenic mHTT might be particularly detrimental in the developing brain, in which physiological HTT function is crucial (Fig. 1).

**Table 1.** Evidence of developmental involvement in HD cases and rodent models

Species	Human/model	Age/disease stage	Phenotypes	Mechanisms	Treatments	References
Human	Aborted fetus	Gestation week 13-16	Developing cortex abnormality: mislocalization of mHTT and junctional complex proteins, abnormal ciliogenesis and changes in mitosis	NA	NA	Barnat et al., 2020
Human	Postmortem brains	Age 36-75	Developmental malformations	NA	NA	Hickman et al., 2021
Human	Postmortem brains	Pre-HD and early pathological grade HD (age 7-30)	Decrease in neuronal fiber density and organization in pyramidal cell layers	Abnormal protein expression in synaptic function and cytoskeletal integrity	NA	DiProspero et al., 2004
Human	Post-mortem brains and imaging	Pre-HD (age ∼44)	Cortical cell loss	Disruption of development (especially in astrocytes and endothelial cells)	NA	Estevez-Fraga et al., 2023
Human	Imaging	Pre-HD (age ∼42)	Abnormal development: smaller intracranial volume	NA	NA	Nopoulos et al., 2011
Human	Imaging	Early stage HD (age ∼42)	Brain sulcus abnormality	NA	NA	Mangin et al., 2020
Human	Imaging	Pre-HD (age ∼39)	Decreased cortical folding complexity	NA	NA	Kubera et al., 2019
Human	Imaging	Age 6-18	Initial striatum and globus pallidus hypertrophy and more rapid volume decline	NA	NA	van der Plas et al., 2019
Human	Imaging and functional assessment	Pre-HD (age ∼41)	Brain volume change, impairment in voluntary motor and oculomotor tasks, cognitive and neuropsychiatric function	NA	NA	Tabrizi et al., 2009
Human	Imaging and functional assessment	Pre-HD (age ∼40)	Decrease in caudate, putamen and globus pallidus volumes consistently correlated with cognitive and motor, but not psychiatric or functional, measures in pre-HD group	NA	NA	Aylward et al., 2013
Human	Imaging and functional assessment	Pre-HD (age ∼41) and early HD (age ∼49)	Significant associations between regional brain atrophy and decline in a range of clinical modalities	NA	NA	Scahill et al., 2013
Human	Cognitive assessment	Age ∼25	Impairments in executive function	NA	NA	Pfalzer et al., 2023
Human	Body fluid, imaging and blood DNA	Age 18-40	Increase in neurofilament light protein, decreased proenkephalin in cerebrospinal fluid, caudate and putamen atrophy, blood somatic CAG repeats expansion ratio increased in HD gene-expanded group	NA	NA	Scahill et al., 2025
Human	Growth parameters	Age ∼13	Weight decrease, body mass index decrease and head circumference decrease in gene-expanded children	NA	NA	Lee et al., 2012
Mouse	Hdh^Q111/Q111^ mouse	STHdh^Q111/Q111^ cell line, E10.5, E14.5 and P21	Spindle orientation of dividing progenitors and cerebral cortex thickness are altered in Hdh^Q111/Q111^ embryos	Alteration of the localization of dynein, NUMA1 and the p150^Glued^ subunit of dynactin to the spindle pole and cell cortex, and of CLIP170 and p150^Glued^ to microtubule plus-ends	The serine/threonine kinase Akt, which regulates HTT function, rescued the spindle misorientation in cultured cells and in mice	Molina-Calavita et al., 2014
Mouse	Hdh^Q111/Q111^ mouse embryo	E12.5-E17.5	Impairment of developmental stem cell-mediated striatal neurogenesis in HD mouse embryos	Sox2, Stat3 and Nanog upregulation in striatal medium spiny neurons	NA	Molero et al., 2009
Mouse	Hdh^Q111/Q111^ mouse embryo	E13.5-E15.5	Developing cortex abnormality: mislocalization of mHTT and junctional complex proteins, defects in neural progenitor cell polarity and differentiation, abnormal ciliogenesis, and changes in mitosis and cell cycle progression	NA	NA	Barnat et al., 2020
Mouse	Hdh^neoQ20/null^ mouse	E15.5 and adult	Severely reduced levels (∼15%) of HTT only during development, subpallial heterotopias, aberrant striatal maturation and deregulation of gliogenesis in embryo stage, as well as late-life striatal and cortical neuronal degeneration, neurological and skeletal muscle alterations, white matter tract impairments and axonal degeneration in adult phase	NA	NA	Arteaga-Bracho et al., 2016
Mouse	Hdh^Q7/Q111^ newborn pup and *in vitro* neuron	P0-P21	Limited growth of layer II/III neurons due to defects in microtubule bundling within the axonal growth cone	Downregulated NUMA1 by miR-124	AntagomiR-124 upregulated NUMA1; epothilone B restored microtubule organization	Capizzi et al., 2022
Mouse	Hdh^Q7/Q111^ mouse	P1-P26 and adult	Reduced dendritic growth and synaptic activity, and increased neuronal excitability in the neonatal cortex during the first postnatal week	NA	Ampakine CX516 treatment restored dendritic arborization and sensorimotor function in HD pups and delayed HD symptoms in the adults	Braz et al., 2022
Mouse	BACHD:CAG-Cre^ERT2^ mouse	P21 and adult	Conditional mHTT expression during development causes striatal neurodegeneration, excitotoxicity, damaged electrophysiological activity, circuit connectivity and plasticity	NA	NA	Molero et al., 2016
Rat	Transgenic HD rat with 51 CAG repeats	P15, P30	Abnormal brain microstructure in brain imaging, reduced and less ordered fiber staining	NA	NA	Blockx et al., 2012

Akt, RAC-alpha serine/threonine-protein kinase; CLIP170, cytoplasmic linker protein 170; E, embryonic day; HD, Huntington's disease; HTT, huntingtin; mHTT, mutant huntingtin; NA, not applicable; NUMA1, nuclear mitotic apparatus protein 1; P, postnatal day; Sox2, SRY-box 2; Stat3, signal transducer and activator of transcription 3.

## Human stem cell models of HD highlight neurodevelopmental aspects of the disease

The establishment of human stem cell models of HD further supports the idea that the disease disrupts human brain development (Kaye et al., 2022; Wiatr et al., 2018) (Table 2). The transcriptional signature of human HD neurons confirms the dysregulation of neurodevelopmental genes (Aguirre et al., 2023 preprint; An et al., 2012; Cohen-Carmon et al., 2020; HD iPSC Consortium, 2017; Mehta et al., 2018; Ring et al., 2015; Świtońska et al., 2018). The presence of mHTT leads to impaired rosette formation and neuronal generation (Mattis et al., 2015; Mehta et al., 2018; Monk et al., 2021; Ruzo et al., 2018; Smith-Geater et al., 2020; Xu et al., 2017). In line with the suggested role of HTT in axonal growth and synaptogenesis (Capizzi et al., 2022; McKinstry et al., 2014), human HD neurons show synaptic dysfunctions (Dinamarca et al., 2022; HD iPSC Consortium, 2012) and defective neuronal branching (Guo et al., 2013; Mehta et al., 2018), with branching progressively decreasing in correlation with increased CAG repeats (Mehta et al., 2018). Human stem cell models of HD recapitulate a dominant-negative effect of mHTT on its wild-type counterpart, suggesting that mHTT hampers the function of wild-type HTT during development (Laundos et al., 2023; Ruzo et al., 2018). Lowering wild-type HTT in human neurons is sufficient to disrupt neuronal maturation and synaptic activity (Louçã et al., 2024), as well as neurite outgrowth capacity (Lisowski et al., 2024).

**Table 2.** Evidence of developmental involvement in human stem cell models

Stem cells	Model	Phenotypes	Mechanisms	Treatments	References
Patient hiPSCs with 71 and 109 CAG repeats	2D hiPSCs	NA	Dysregulated transcripts in DNA damage, apoptosis, cell polarization, transcription regulators of development; altered TP53 and ZFP30 protein expression	NA	Świtońska et al., 2018
Patient hiPSCs with 72 CAG repeats	2D NSCs	Increased susceptibility to cell death and altered mitochondrial bioenergetics	Dysregulated pathogenic HD signaling pathways (cadherin, TGF-β, BDNF, SMAD and caspase activation)	Replacement of the expanded CAG repeat with a normal repeat reversed disease phenotypes	An et al., 2012; Ring et al., 2015
Patient hiPSCs with 77, 109 and 180 CAG repeats	2D NPCs	Increased death following BDNF withdrawal	TrkB receptor downregulation and increased glutamate toxicity caused by upregulated NR2B	Activating TrkB and blocking glutamate signaling reversed the cell death phenotype	Mattis et al., 2015
Patient hiPSCs with CAG180	2D NPCs and neural cells	Impaired neural rosette formation, increased susceptibility to growth factor withdrawal and deficits in mitochondrial respiration in HD hiPSC-derived neural cells	Gene expression differences including altered *CHCHD2* expression	Correction of HTT mutation reversed HD phenotypes	Xu et al., 2017
Patient hiPSCs with 46, 53, 66, 71 and 109 CAG repeats	2D NSCs and striatal neurons	Persistent cyclin D1^+^ NSC population in HD striatal neurons	Upregulation of cell-cycle-related genes and transcription factors	Inhibition of the WNT signaling pathway abrogates NSC populations in HD neuronal cultures	Smith-Geater et al., 2020
Patient hiPSCs with 41Q, 43Q, 44Q, 57Q	2D striatal neurons	Ubiquitinated polyglutamine aggregates HD striatal neurons, impaired neuronal maturation	Reduced BDNF expression	NA	Monk et al., 2021
Patient hiPSC with 46, 53, 60, 109 CAG repeats	2D neural cells	Increased susceptibility to BDNF withdrawal, cell death, longer neurite-like process	Upregulation of genes in glutamate and GABA signaling, axonal guidance, and calcium influx; altered epigenetic program	Isx-9 treatment improves CAG repeat-associated phenotypes	HD iPSC Consortium, 2017
Edited hESCs lines with 20, 22, 42, 48, 56, 67, 72 CAG repeats	2D neural cells	Giant multinucleated hESC-generated telencephalic neurons at an abundance directly proportional to CAG repeat length	Chromosomal instability and failed cytokinesis over multiple rounds of DNA replication	NA	Ruzo et al., 2018
Edited hiPSCs with 72Q	2D neurons	Abnormal synapse and delayed neural maturation in hiPSC-derived neurons	NA	NA	Dinamarca et al., 2022
Patient hiPSCs with 77, 109 and 180 CAG repeats	2D cortical neurons	Delayed functional maturation, shorter neuritic extensions in HD neurons	Altered transcriptomics in neural development	NA	Mehta et al., 2018
Patient hiPSCs with 41, 45, 46 and 48 CAG repeats	2D striatal GABAergic neurons	NA	Altered IGF1 and genes involved in neurogenesis and nervous system development under progerin treatment	NA	Cohen-Carmon et al., 2020
Edited hESCs lines with 72 CAG expansion	2D hESCs and 3D neuruloids	Aberrant impaired polarity and receptor mislocalization in HD hESCs, and failed compaction of the central neural ectodermal domain and in the reduction of the neural crest linage in HD neuruloids	NA	Wild-type HTT overexpression partially reversed the HD phenotypes	Laundos et al., 2023
Patient hiPSCs with 60, 109 and 180 CAG repeats	2D striatal, cortical neurons and 3D cerebral organoids	Faulty neuronal determination and cell polarization in HD organoids	NA	mHTT repressor and GI254023X treatment recovered striatal identity	Conforti et al., 2018
Patient hiPSCs with 18Q, 71Q and 109Q	2D NSC and 3D forebrain organoids	Dysregulated cell cycle in HD NSC and premature neuronal differentiation in HD forebrain organoids	Increased activity of the ATM–p53 pathway	ATM antagonists partially rescued the blunted neuroepithelial progenitor expansion	Zhang et al., 2019 preprint
Edited hiPSCs with 70Q and patient hiPSCs with 44Q, 58Q, 180Q	2D NPCs, neurons, and 3D cerebral, cortical and midbrain organoids	Aberrant development of cerebral organoids with loss of neural progenitor organization	Downregulation of CHCHD2, increase in mitochondrial integrated stress response, defective mitochondrial morpho-dynamics and aberrant metabolic programming	CHCHD2 overexpression or polyQ removal corrected mitochondrial defects	Lisowski et al., 2024
Edited hESCs with 56 and 72 CAG expansions	3D neuruloids	Abnormal neuruloid morphogenesis	Downregulation of WNT/PCP pathway, cytoskeleton-associated genes and actin–myosin contraction genes	NA	Haremaki et al., 2019
Edited hESCs with 48, 56 and 72 CAG repeats	3D mono-cultures and mosaic telencephalic organoids	Altered differentiation pattern, self-organization and ventral maturation in mono-culture HD telencephalic organoids	Weakened intercellular communication in HD	HD cells, especially ventral neurons, recover maturation and fate determination when grown with control cells in mosaic HD telencephalic organoids	Galimberti et al., 2024
Patient hiPSCs with 55 and 59 CAG expansions	3D striatal organoids	Smaller size and impaired differentiation of striatal organoids, without MSN maturation defect	NA	NA	Chen et al., 2022
Patient hiPSCs with 75 CAG repeats	3D striatal organoids	More neuron death in striatal organoids	HSF1 accumulated in mitochondria causing mitochondrial fission and mtDNA deletion	The unique peptide inhibitor DH1 suppressed localization of HSF1 in mitochondria, mitochondrial dysfunction, and cell death	Liu et al., 2022
Patient hiPSCs with 55 and 59 CAG repeats	3D cortical, and cortico-striatal assembloids	Deficient progenitor proliferation, premature neurogenesis, deficiency of cortical projection neurons and laminations, delayed maturation of postmitotic neurons, aberrantly early HD cortical projections targeting striatal organoids	Deficient Golgi apparatus, clathrin^+^ vesicles and junctional complex. Endogenous mHTT lowered Golgi recruiting ARF1	NA	Liu et al., 2024a,b
Patient hiPSCs with 75 CAG repeats and two patient hiPSCs not specified	3D striatal, midbrain organoid and striato-nigral assembloids	Reciprocal projection defects in striatum-like and midbrain substantial nigra-like assembloids	NA	BDNF rescued reciprocal projection and calcium signaling	Wu et al., 2024

ARF1, ADP-ribosylation factor; ATM, ataxia telangiectasia mutated; BDNF, brain-derived neurotrophic factor; CHCHD2, coiled-coil-helix-coiled-coil-helix domain containing 2; GABA, gamma-aminobutyric acid; HD, Huntington's disease; hESC, human embryonic stem cell; hiPSC, human induced pluripotent stem cell; HSF1, heat shock transcription factor 1; HTT, huntingtin; IGF1, insulin-like growth factor 1; Isx-9, isoxazole 9; mHTT, mutant huntingtin; MSN, medium spiny neuron; mtDNA, mitochondrial DNA; NA, not applicable; NR2B, N-methyl D-aspartate receptor subtype 2B (also known as GRIN2B); NSC, neural stem cell; NPC, neural progenitor cell; ; PCP, planar cell polarity; SMAD, mothers against decapentaplegic homolog; TP53, tumor protein P53; TrkB, tropomyosin receptor kinase B (also known as NTRK2); TGF-β, transforming growth factor beta; ZFP30, zinc finger protein 30.

Recent advances in induced pluripotent stem cell (iPSC)-derived three-dimensional (3D) culture allow the generation of brain organoids, which may recapitulate key aspects of the complex brain architecture, including cellular composition and interactions (Birtele et al., 2025). Organoids representative of distinct brain regions (Del Dosso et al., 2020) can also be merged to investigate neuronal projections and circuit formation (Onesto et al., 2024). Currently, brain organoids lack non-ectodermal cells, such as vasculature and microglia, reducing their maturation and their utility in studying neuroinflammation (Andrews and Kriegstein, 2022). Furthermore, as they reflect fetal or neonatal stages of development, they cannot address aging-related aspects (Andrews and Kriegstein, 2022). Nonetheless, these complex model systems could be instrumental in providing insights into the impact of wild-type HTT and mHTT on human brain development.

HD cortical organoids show aberrant neuronal determination (Conforti et al., 2018). Striatal assembloids generated by fusing striatal organoids with cortical or midbrain organoids to investigate striatal connectivity (Chen et al., 2022) exhibit defective SPN projections when carrying mHTT (Wu et al., 2024). HD neuruloids modeling neural tube formation develop alterations (Haremaki et al., 2019), potentially suggesting that mHTT disrupts early neural commitment. Accordingly, loss of neural progenitor organization occurs within mHTT-carrying telencephalic organoids (Galimberti et al., 2024), cortical organoids (Lisowski et al., 2024) and whole-brain cerebral organoids (Zhang et al., 2019 preprint). Defective junctional complexes between neighboring progenitors observed in HD cortical organoids (Lisowski et al., 2024; Liu et al., 2024b) could contribute to disrupt spatiotemporal corticogenesis (Liu et al., 2024b).

Altogether, these findings show that human stem cell models of HD demonstrate impairment of central nervous system development (Table 2).

Accounting for the impact of HD on neurodevelopment could lead to the development of innovative therapeutic strategies

## Leveraging compensatory mechanisms for HD therapeutics

Accounting for the impact of HD on neurodevelopment could lead to the development of innovative therapeutic strategies (Humbert, 2010; Mehler and Gokhan, 2000; van der Plas et al., 2020) (Fig. 1). Indeed, early correction of circuit defects in HD mice can delay the brain pathology (Braz et al., 2022). At the same time, this shift in focus brings several challenges.

A key question relates to the compensatory mechanisms that allow the brain of individuals with HD to preserve functionality for a long time. Such mechanisms could influence whether CAG repeats present in the genomes of individuals who may develop disease symptoms are further expanded in certain somatic cells owing to genomic instability (Handsaker et al., 2025). In mice, early defects in SPN projection are normalized during late development (Lebouc et al., 2025), indicating the existence of processes that could possibly be preserved or enhanced therapeutically to postpone the disease onset. However, it is not trivial to distinguish disease-driving alterations from strategies that the body has established to compensate for the primary genetic defect. Attempts to untangle these processes in flies and human HD neurons have highlighted the modulation of calcium and synaptic function as being involved in this compensation (Al-Ramahi et al., 2018). Additional efforts should be undertaken to uncover compensatory mechanisms and distinguish them from driver mechanisms. This is crucial when developing therapies in order to avoid disrupting pathways that have already been effectively counteracted by the body.

Compensatory mechanisms may include cell-autonomous pathways that directly affect the behavior of a cell (Berto et al., 2025). For example, early dysregulation of the gene coiled-coil-helix-coiled-coil-helix domain containing 2 (*CHCHD2*) can be seen in HD mice, human HD neurons (Liu et al., 2024a) and HD brain organoids (Lisowski et al., 2024; Liu et al., 2024a). CHCHD2 regulates mitochondrial function and stress responses (Ruan et al., 2022; Zhou et al., 2020) and may thus help maintain a cell-autonomous, compensatory, protective response in bioenergetically impaired HD neurons. As oxidative damage may contribute to somatic CAG expansion (Kovtun et al., 2007), measures preserving redox balance and mitochondrial fitness could be beneficial in maintaining a compensated state.

Non-cell-autonomous mechanisms, i.e. involving neighboring cells or extracellular signals (Berto et al., 2025), might also contribute to preserving brain function in HD. For example, abnormal development of some brain regions may be compensated for by other brain regions (Catlin and Schaner Tooley, 2024; van der Plas et al., 2020). The cerebellum, which appears spared in early HD cases (Aylward, 2007), might compensate for faulty striatal function by preventing involuntary movements (van der Plas et al., 2020). However, this compensatory rerouting of circuitry might not persist over time, as the volume of the cerebellum decreases in older HD cases (Sarappa et al., 2017; Tereshchenko et al., 2019). Brain organoids grown as mosaic co-culture of healthy and HD cells demonstrate that the faulty developmental trajectory of HD cells can be restored by the presence of healthy cells. This further supports the existence of non-cell-autonomous compensatory mechanisms that may be explored as potential interventions (Galimberti et al., 2024). Additional roles in this compensation may be played by glial cells (Benraiss et al., 2016) or by transfer of mitochondria, which could modulate energy homeostasis (Geng et al., 2023) and inflammation (Joshi et al., 2019).

… we propose that harnessing compensatory pathways promoting mitochondrial fitness could represent a therapeutic strategy for HD

## Targeting HD at the early stage to preserve mitochondrial fitness

Recent findings highlight that mitochondrial function is crucial for human brain development (Brunetti et al., 2021; Iwata et al., 2023, 2020). Mitochondrial defects are present in human stem cell-derived HD neurons (Chae et al., 2012; Feyeux et al., 2012; HD iPSC Consortium, 2012, 2020; Lopes et al., 2020; McQuade et al., 2014; Nekrasov and Kiselev, 2018; Szlachcic et al., 2015; Xu et al., 2017). Modulating genes such as *CHCHD2* that regulate mitochondrial stress could be beneficial in HD neural cells (Lisowski et al., 2024). Hence, we propose that harnessing compensatory pathways promoting mitochondrial fitness could represent a therapeutic strategy for HD (Fig. 1).

Metabolic disturbances are known to occur in individuals with HD who may experience a state of catabolism, in which there is increased energy expenditure and weight loss irrespective of caloric intake (Dubinsky, 2017; Gilbert, 2009; Ogilvie et al., 2021; van der Burg et al., 2017). Metabolic impairment – including reduced glucose metabolism in the brain (Grafton et al., 1992), defects in cholesterol and fatty acid metabolism (Block et al., 2010), and skeletal muscle wastage (Zielonka et al., 2014) – can occur years before the neuropathological manifestations (Singh and Agrawal, 2022). Hence, it is possible that metabolic imbalance and related oxidative stress could contribute to the loss of compensation in HD over time.

In agreement with this view, several treatment options put forward in HD models may work through improving mitochondrial health and preventing cellular stress. For example, inhibition of DRP1 (also known as DNM1L), a mitochondrial fission protein that directly binds mHTT (Song et al., 2011), ameliorates neurite outgrowth capacity and survival in human HD neurons and reduces mortality in HD mice (Guo et al., 2013). Ataxia-telangiectasia mutated (ATM), a key signaling molecule activated in response to oxidative stress and DNA damage, may be hyperactive in HD. Reduction of ATM lowers mHTT toxicity in HD models (Lu et al., 2014) and improves neural progenitor expansion in HD brain organoids (Zhang et al., 2019 preprint). The oxidative-damage-related cytokine, N6-furfuryladenine (N6FFA), can reverse HD-like phenotypes in mice (Bowie et al., 2018). The fatty acid synthesis inhibitor cerulenin ameliorates the disease-associated transcriptional signature of human SPNs (Aguirre et al., 2023 preprint). Inhibition of mitochondrially located heat shock transcription factor 1 (HSF1) can restore mitochondrial morphology and neuronal outgrowth in HD striatal organoids and improves the behavioral function of HD mice (Liu et al., 2022).

## Conclusions

Here, we have discussed how understanding the impact of neurodevelopmental alterations in HD pathogenesis could lead to the establishment of innovative interventions. Potential therapeutics could be developed by harnessing endogenous compensatory mechanisms. One such strategy could involve preserving mitochondrial fitness to avoid DNA damage and cellular stress, which could otherwise exacerbate somatic CAG expansion in susceptible brain regions over time (Jonson et al., 2013; Kovtun et al., 2007; Wang et al., 2025). Several challenges still lie ahead, including better understanding the potential early intervention strategies and defining specific therapeutic windows. Nonetheless, the compelling evidence indicating the importance of brain development in HD pathogenesis should prompt us to determine whether it may be possible to target these early defects to delay or even prevent the neurological deterioration in affected individuals.

## Supplementary Material

10.1242/dmm.052510_sup1 Supplementary information
