# Supplementary material for: Brain organoid models of Huntington's disease shift the focus towards neurodevelopment
Source: Dis Model Mech. 2025 Oct 28;18(10):dmm052510. doi: 10.1242/dmm.052510 (PMC12598923; doi:10.1242/dmm.052510)
Supplement: Supplementary information [file dmm-18-052510-s1.pdf]

Table S1. Evidence of developmental involvement in HD cases and rodent models

Species	Human / Model	Age / Disease stage	Phenotypes	Mechanisms	Treatments	References
Human	Aborted fetus	Gestation week 13 - 16	Developing cortex abnormality: mislocalization of mutant huntingtin and junctional complex proteins, abnormal ciliogenesis, and changes in mitosis	NA	NA	Barnat et al., 2020
Human	Postmortem brains	Age 36-75	Developmental malformations	NA	NA	Hickman et al., 2021
Human	Postmortem brains	Pre-HD and early pathological grade HD (age 7-30)	Decrease in neuronal fiber density and organization in pyramidal cell layers	Abnormal protein expression in synaptic function and cytoskeletal integrity	NA	DiProspero et al., 2004
Human	Post-mortem brains and imaging	Pre-HD (age ~44)	Cortical cell loss	Developmental genes disruption (especially in astrocytes and endothelial cells)	NA	Estevez-Fraga et al., 2023
Human	Imaging	Pre-HD (age ~42)	Abnormal development: Smaller intracranial volume	NA	NA	Nopoulos et al., 2011
Human	Imaging	Early stage HD (age ~42)	Brain sulcus abnormality	NA	NA	Mangin et al., 2020
Human	Imaging	Pre-HD (age ~39)	Decreased cortical folding complexity	NA	NA	Kubera et al., 2019
Human	Imaging	Age 6–18	Initial striatum and globus pallidus hypertrophy and more rapid volume decline.	NA	NA	van der Plas et al., 2019
Human	Imaging and functional assessment	Pre-HD (age ~41)	Brain volume change, impairment in voluntary motor, and oculomotor tasks, cognitive, and neuropsychiatric function	NA	NA	Tabrizi et al., 2009
Human	Imaging and	Pre-HD (age	Caudate, putamen, and globus	NA	NA	Aylward et

	functional assessment	~40)	pallidus volumes↓ consistently correlated with cognitive and motor, but not psychiatric or functional measures in preHD group			al., 2013
Human	Imaging and functional assessment	Pre-HD (age ~41) and early HD (age ~49)	significant associations between regional brain atrophy and decline in a range of clinical modalities	NA	NA	Scahill et al., 2013
Human	Cognitive assessment	Age ~25	Impairments in executive function	NA	NA	Pfalzer et al., 2023
Human	Body fluid, imaging and blood DNA	Age 18-40	neurofilament light protein ↑, proenkephalin↓ in cerebrospinal fluid, caudate and putamen atrophy, blood somatic CAG repeats expansion ratio ↑ in HD gene expanded group	NA	NA	Scahill et al., 2025
Human	Growth parameters	Age ~13	weight↓, body mass index↓, and head circumference↓ in gene-expanded children	NA	NA	Lee et al., 2012
Mouse	$Hdh^{Q111/Q111}$ mouse	$STHdh^{Q111/Q111}$ cell line, E10.5, E14.5 days mouse and postnatal 21 days	Spindle orientation of dividing progenitors and cerebral cortex thickness are altered in $Hdh^{Q111/Q111}$ embryos	Alteration of the localization of dynein, NuMA, and the $p150^{Glued}$ subunit of dynactin to the spindle pole and cell cortex, and of CLIP170 and $p150^{Glued}$ to microtubule plus-ends	The serine/threonine kinase Akt, which regulates HTT function, rescued the spindle misorientation in cultured cells and in mice	Molina-Calavita et al., 2014
Mouse	$Hdh^{Q111/Q111}$ mouse embryo	E12.5 - 17.5 days	Impairment of developmental stem cell-mediated striatal neurogenesis in HD mouse embryos	Sox2, Stat3 and Nanog upregulation in striatal medium spiny neuron	NA	Molero et al., 2009
Mouse	$Hdh^{Q111/Q111}$ mouse embryo	E13.5 - 15.5 days	Developing cortex abnormality: mislocalization of mutant huntingtin and junctional complex	NA	NA	Barnat et al., 2020

Disease Models & Mechanisms • Supplementary information

			proteins, defects in neural progenitor cell polarity and differentiation, abnormal ciliogenesis, and changes in mitosis and cell cycle progression.			
Mouse	$Hdh^{neoQ20/null}$ mouse	E15.5 days and adult mice	$Hdh^{neoQ20/null}$ mouse, with severely reduced levels (~15%) of huntingtin only during development, showed subpallial heterotopias, aberrant striatal maturation and deregulation of gliogenesis in embryo stage, as well as late-life striatal and cortical neuronal degeneration, neurological and skeletal muscle alterations, white matter tract impairments and axonal degeneration in adult phase	NA	NA	Arteaga-Bracho et al., 2016
Mouse	$Hdh^{Q7/Q111}$ newborn pup and *in vitro* neuron	Postnatal 0-21 days	Limited growth of layer II/III neurons due to defects in microtubule bundling within the axonal growth cone	Downregulated NUMA1 by miR-124	AntagomiR-124: upregulated NUMA1; epothilone B: restored microtubule organization	Capizzi et al., 2022
Mouse	$Hdh^{Q7/Q111}$ mouse	Postnatal 1-26 days and adult	Reduced dendritic growth, synaptic activity, and increased neuronal excitability in the neonatal cortex during the first postnatal week	NA	Ampakine CX516 treatment restored dendritic arborization and sensorimotor function in HD pups and delayed HD symptoms in the adults	Braz et al., 2022
Mouse	BACHD:CAG-Cre^{ERT2} mouse	Postnatal 21 days and adult	Conditional mHTT expression during development causes striatal neurodegeneration, excitotoxicity, damaged	NA	NA	Molero et al., 2016

Disease Models & Mechanisms • Supplementary information

			electrophysiological activity, circuit connectivity and plasticity, motor function in adults			
Rat	Transgenic HD rat with 51 CAG repeats	Postnatal 15, 30 days	Abnormal brain microstructure in brain imaging, reduced and less ordered fiber staining	NA	NA	Blockx et al., 2012

Abbreviations: *HD, Huntington's Disease; NA, not applicable; HTT, huntingtin; mHTT, mutant huntingtin; NUMA1, nuclear mitotic apparatus protein 1; CLIP170, Cytoplasmic linker Protein 170; sox2, SRY-box 2; stat3, Signal transducer and activator of transcription 3.*

Disease Models & Mechanisms • Supplementary information

References

Arteaga-Bracho, E. E., Gulinello, M., Winchester, M. L., Pichamoorthy, N., Petronglo, J. R., Zambrano, A. D., Inocencio, J., De Jesus, C. D., Louie, J. O., Gokhan, S. et al. (2016). Postnatal and adult consequences of loss of huntingtin during development: Implications for Huntington's disease. *Neurobiol Dis* **96**, 144-155.

Aylward, E. H., Harrington, D. L., Mills, J. A., Nopoulos, P. C., Ross, C. A., Long, J. D., Liu, D., Westervelt, H. K. and Paulsen, J. S. (2013). Regional atrophy associated with cognitive and motor function in prodromal Huntington disease. *J Huntingtons Dis* **2**, 477-89.

Barnat, M., Capizzi, M., Aparicio, E., Boluda, S., Wennagel, D., Kacher, R., Kassem, R., Lenoir, S., Agasse, F., Braz, B. Y. et al. (2020). Huntington's disease alters human neurodevelopment. *Science* **369**, 787-793.

Blockx, I., De Groof, G., Verhoye, M., Van Audekerke, J., Raber, K., Poot, D., Sijbers, J., Osmand, A. P., Von Hörsten, S. and Van der Linden, A. (2012). Microstructural changes observed with DKI in a transgenic Huntington rat model: evidence for abnormal neurodevelopment. *Neuroimage* **59**, 957-67.

Braz, B. Y., Wennagel, D., Ratié, L., de Souza, D. A. R., Deloulme, J. C., Barbier, E. L., Buisson, A., Lanté, F. and Humbert, S. (2022). Treating early postnatal circuit defect delays Huntington's disease onset and pathology in mice. *Science* **377**, eabq5011.

Capizzi, M., Carpentier, R., Denarier, E., Adrait, A., Kassem, R., Mapelli, M., Couté, Y. and Humbert, S. (2022). Developmental defects in Huntington's disease show that axonal growth and microtubule reorganization require NUMA1. *Neuron* **110**, 36-50.e5.

DiProspero, N. A., Chen, E. Y., Charles, V., Plomann, M., Kordower, J. H. and Tagle, D. A. (2004). Early changes in Huntington's disease patient brains involve alterations in cytoskeletal and synaptic elements. *J Neurocytol* **33**, 517-33.

Estevez-Fraga, C., Altmann, A., Parker, C. S., Scahill, R. I., Costa, B., Chen, Z., Manzoni, C., Zarkali, A., Durr, A., Roos, R. A. C. et al. (2023). Genetic topography and cortical cell loss in Huntington's disease link development and neurodegeneration. *Brain* **146**, 4532-4546.

Hickman, R. A., Faust, P. L., Rosenblum, M. K., Marder, K., Mehler, M. F. and Vonsattel, J. P. (2021). Developmental malformations in Huntington disease: neuropathologic evidence of focal neuronal migration defects in a subset of adult brains. *Acta Neuropathol* **141**, 399-413.

Kubera, K. M., Schmitgen, M. M., Hirjak, D., Wolf, R. C. and Orth, M. (2019). Cortical neurodevelopment in pre-manifest Huntington's disease. *Neuroimage Clin* **23**, 101913.

Lee, J. K., Mathews, K., Schlaggar, B., Perlmutter, J., Paulsen, J. S., Epping, E., Burmeister, L. and Nopoulos, P. (2012). Measures of growth in children at risk for Huntington disease. *Neurology* **79**, 668-74.

Mangin, J. F., Rivière, D., Duchesnay, E., Cointepas, Y., Gaura, V., Verny, C., Damier, P., Krystkowiak, P., Bachoud-Lévi, A. C., Hantraye, P. et al. (2020). Neocortical morphometry in Huntington's disease: Indication of the coexistence of abnormal neurodevelopmental and neurodegenerative processes. *Neuroimage Clin* **26**, 102211.

Molero, A. E., Arteaga-Bracho, E. E., Chen, C. H., Gulinello, M., Winchester, M. L., Pichamoorthy, N., Gokhan, S., Khodakhah, K. and Mehler, M. F. (2016). Selective expression of mutant huntingtin during development recapitulates characteristic features of Huntington's disease. *Proc Natl Acad Sci U S A* **113**, 5736-41.

Molero, A. E., Gokhan, S., Gonzalez, S., Feig, J. L., Alexandre, L. C. and Mehler, M. F. (2009). Impairment of developmental stem cell-mediated striatal neurogenesis and pluripotency genes in a knock-in model of Huntington's disease. *Proc Natl Acad Sci U S A* **106**, 21900-5.

Disease Models & Mechanisms • Supplementary information

Molina-Calavita, M., Barnat, M., Elias, S., Aparicio, E., Piel, M. and Humbert, S. (2014). Mutant huntingtin affects cortical progenitor cell division and development of the mouse neocortex. *J Neurosci* **34**, 10034-40.

Nopoulos, P. C., Aylward, E. H., Ross, C. A., Mills, J. A., Langbehn, D. R., Johnson, H. J., Magnotta, V. A., Pierson, R. K., Beglinger, L. J., Nance, M. A. et al. (2011). Smaller intracranial volume in prodromal Huntington's disease: evidence for abnormal neurodevelopment. *Brain* **134**, 137-42.

Pfalzer, A. C., Watson, K. H., Ciriegio, A. E., Hale, L., Diehl, S., McDonell, K. E., Vnencak-Jones, C., Huitz, E., Snow, A., Roth, M. C. et al. (2023). Impairments to executive function in emerging adults with Huntington disease. *J Neurol Neurosurg Psychiatry* **94**, 130-135.

Scahill, R. I., Farag, M., Murphy, M. J., Hobbs, N. Z., Leocadi, M., Langley, C., Knights, H., Ciosi, M., Fayer, K., Nakajima, M. et al. (2025). Somatic CAG repeat expansion in blood associates with biomarkers of neurodegeneration in Huntington's disease decades before clinical motor diagnosis. *Nat Med*.

Scahill, R. I., Hobbs, N. Z., Say, M. J., Bechtel, N., Henley, S. M., Hyare, H., Langbehn, D. R., Jones, R., Leavitt, B. R., Roos, R. A. et al. (2013). Clinical impairment in premanifest and early Huntington's disease is associated with regionally specific atrophy. *Hum Brain Mapp* **34**, 519-29.

Tabrizi, S. J., Langbehn, D. R., Leavitt, B. R., Roos, R. A., Durr, A., Craufurd, D., Kennard, C., Hicks, S. L., Fox, N. C., Scahill, R. I. et al. (2009). Biological and clinical manifestations of Huntington's disease in the longitudinal TRACK-HD study: cross-sectional analysis of baseline data. *Lancet Neurol* **8**, 791-801.

van der Plas, E., Langbehn, D. R., Conrad, A. L., Koscik, T. R., Tereshchenko, A., Epping, E. A., Magnotta, V. A. and Nopoulos, P. C. (2019). Abnormal brain development in child and adolescent carriers of mutant huntingtin. *Neurology* **93**, e1021-e1030.

Disease Models & Mechanisms • Supplementary information

Table S2. Evidence of developmental involvement in human stem cell models

Stem cells	Model	Phenotypes	Mechanisms	Treatments	References
Patient iPSCs with 71 and 109 CAG repeats	2D iPSCs	NA	Dysregulated transcripts in DNA damage, apoptosis, cell polarization, transcription regulators of development. Altered TP53 and ZFP30 protein expression	NA	Świtońska et al., 2018
Patient iPSCs with 72 CAG repeats	2D NSCs	Increased susceptibility to cell death and altered mitochondrial bioenergetics	Dysregulated pathogenic HD signaling pathways (cadherin, TGF-b, BDNF, SMAD, and caspase activation)	Replacement of the expanded CAG repeat with a normal repeat reversed disease phenotypes	An et al., 2012; Ring et al., 2015
Patient iPSCs with 77, 109 and 180 CAG repeats	2D NPCs	Increased death following BDNF withdrawal	TrkB receptor downregulation and Increased glutamate toxicity caused by up-regulated NR2B	Activate TrkB and blocking glutamate signaling reversed the cell death phenotype	Mattis et al., 2015
Patient iPSCs with CAG180	2D NPCs and neural cells	Impaired neural rosette formation, increased susceptibility to growth factor withdrawal, and deficits in mitochondrial respiration in HD hiPSC-derived neural cells	Gene expression differences including altered CHCHD2 expression	Correction of HTT mutation reversed HD phenotypes	Xu et al., 2017

Patient iPSCs with 46, 53, 66, 71 and 109 CAG repeats	2D NSCs and striatal neurons	Persistent cyclin D1+ NSC population in HD striatal neurons	Upregulation of cell-cycle-related genes and transcription factors	Inhibition of the WNT signaling pathway abrogates NSC populations in HD neuronal cultures	Smith-Geater et al., 2020
Patient iPSCs with 41Q, 43Q, 44Q, 57Q	2D striatal neurons	Ubiquitinated polyglutamine aggregates HD striatal neurons, impaired neuronal maturation	Reduced BDNF expression	NA	Monk et al., 2021
Patient iPSC with 46, 53, 60, 109 CAG repeats	2D neural cells	Increased susceptibility to BDNF withdraw, cell death, longer neurite-like process	Upregulation of genes in glutamate and GABA signaling, axonal guidance, and calcium influx; altered epigenetic program	Isx-9 treatment improves CAG repeat-associated phenotypes	HD iPSC Consortium, 2017
Edited hESCs lines with 20, 22, 42, 48, 56, 67, 72 CAG repeats	2D neural cells	Giant multinucleated hESC-generated telencephalic neurons at an abundance directly proportional to CAG repeat length	Chromosomal instability and failed cytokinesis over multiple rounds of DNA replication	NA	Ruzo et al., 2018
Edited iPSCs with 72Q	2D neurons	Abnormal synapse and delayed neural maturation in hiPSC-derived neurons	NA	NA	Dinamarca et al., 2022
Patient iPSCs with 77, 109 and 180 CAG	2D cortical neurons	Delayed functional maturation, shorter neuritic extensions in	Altered transcriptomics in neural development	NA	Mehta et al., 2018

Disease Models & Mechanisms • Supplementary information

repeats		HD neurons			
Patient iPSCs with 41, 45, 46 and 48 CAG repeats	2D striatal GABAergic neurons	NA	Altered IGF1 and genes involved in neurogenesis and nervous system development under Progerin treatment	NA	Cohen-Carmon et al., 2020
Edited hESCs lines with 72 CAG expansion	2D hESCs and 3D neuruloids	Aberrant impaired polarity and receptor mislocalization in HD hESCs, and failed compaction of the central neural ectodermal domain and in the reduction of the neural crest linage in HD neuruloids	NA	Wildtype HTT overexpression partially reversed the HD phenotypes	Laundos et al., 2023
Patient iPSCs with 60, 109, and 180 CAG repeats	2D striatal, cortical neurons and 3D cerebral organoids	Faulty neuronal determination and cell polarization in HD organoids	NA	mHTT repressor and GI254023X treatment recovered striatal identity	Conforti et al., 2018
Patient iPSCs with 18Q, 71Q, and 109Q	2D NSC and 3D forebrain organoids	Dysregulated cell cycle in HD NSC and premature neuronal differentiation in HD forebrain organoids	Increased activity of the ATM-p53 pathway	ATM antagonists partially rescued the blunted neuroepithelial progenitor expansion	Zhang et al., 2019
Edited iPSCs with 70Q and patient iPSCs with 44Q,	2D NPCs, neurons, and 3D cerebral, cortical, and	Aberrant development of cerebral organoids with loss of neural progenitor organization	Downregulation of CHCHD2, increase in mitochondrial integrated stress	CHCHD2 overexpression or polyQ removal corrected	Lisowski et al., 2024

Disease Models & Mechanisms • Supplementary information

58Q, 180Q	midbrain organoids		response, defective mitochondrial morpho-dynamics and aberrant metabolic programming	mitochondrial defects	
Edited hESCs with 56 and 72 CAG expansions	3D neuruloids	Abnormal neuruloid morphogenesis	Wnt/PCP pathway, cytoskeleton-associated genes, and actin–myosin contraction genes downregulation	NA	Haremaki et al., 2019
Edited hESCs with 48, 56 and 72 CAG repeats	3D mono-cultures and mosaic telencephalic organoids	Altered differentiation pattern, self-organization and ventral maturation in mono-culture HD telencephalic organoids	Weakened intercellular communication in HD	HD cells, especially ventral neurons, recover maturation and fate determination when grown with control cells in mosaic HD telencephalic organoids	Galimberti et al., 2024
Patient iPSCs with 55 and 59 CAG expansions	3D striatal organoids	Smaller size and impaired differentiation of striatal organoid, without MSNs maturation defect	NA	NA	Chen et al., 2022
Patient iPSCs with 75 CAG repeats	3D striatal organoids	More neuron death in striatal organoids	HSF1 accumulated in mitochondria causing mitochondrial fission and mtDNA deletion	DH1 suppressed localization of HSF1 in mitochondria, mitochondria dysfunction, and cell	Liu et al., 2022

Disease Models & Mechanisms • Supplementary information

				death	
Patient iPSCs with 55 and 59 CAG repeats	3D cortical, and cortico-striatal assembloids	Deficient progenitor proliferation, premature neurogenesis, deficiency of cortical projection neurons and laminations, delayed maturation of postmitotic neurons, aberrantly early HD cortical projections targeting striatal organoids	Deficient Golgi apparatus, clathrin+ vesicles and the junctional complex. Endogenous mHTT lowered Golgi recruiting ARF1	NA	Liu et al., 2024
Patient hiPSCs with 75 CAG repeats and two patient iPSCs not specified	3D striatal, midbrain organoid and striato-nigral assembloids	Reciprocal projection defects in striatum-like and midbrain substantial nigra-like assembloids	NA	Brain-derived neurotrophic factor rescued reciprocal projection and calcium signaling	Wu et al., 2024

Abbreviations: *HD, Huntington's Disease; NA, not applicable; HTT, huntingtin; mHTT, mutant huntingtin; NSC, Neural stem cell; NPC, Neural progenitor cell; hESC, Human embryonic stem cell; iPSC, Human induced pluripotent stem cell; MSN, Medium spiny neuron; TP53, Tumor protein P53; ZFP30, Zinc Finger Protein 30; TGF-b, Transforming Growth Factor beta; BDNF, Brain-Derived Neurotrophic Factor; SMAD, Mothers against decapentaplegic homolog; TrkB, Tropomyosin receptor kinase B; NR2B, N-methyl D-aspartate receptor subtype 2B; CHCHD2, Coiled-coil-helix-coiled-coil-helix domain containing 2; GABA, Gamma-aminobutyric acid; IGF1, Insulin-like Growth Factor 1; ATM, Ataxia telangiectasia mutated; PCP, Planar cell polarity; HSF1, Heat shock transcription factor 1; ARF1, ADP-ribosylation factor*

References

An, M. C., Zhang, N., Scott, G., Montoro, D., Wittkop, T., Mooney, S., Melov, S. and Ellerby, L. M. (2012). Genetic correction of Huntington's disease phenotypes in induced pluripotent stem cells. *Cell Stem Cell* **11**, 253-63.

Chen, X., Saiyin, H., Liu, Y., Wang, Y., Li, X., Ji, R. and Ma, L. (2022). Human striatal organoids derived from pluripotent stem cells recapitulate striatal development and compartments. *PLoS Biol* **20**, e3001868.

Cohen-Carmon, D., Sorek, M., Lerner, V., Divya, M. S., Nissim-Rafinia, M., Yarom, Y. and Meshorer, E. (2020). Progerin-Induced Transcriptional Changes in Huntington's Disease Human Pluripotent Stem Cell-Derived Neurons. *Mol Neurobiol* **57**, 1768-1777.

Conforti, P., Besusso, D., Bocchi, V. D., Faedo, A., Cesana, E., Rossetti, G., Ranzani, V., Svendsen, C. N., Thompson, L. M., Toselli, M. et al. (2018). Faulty neuronal determination and cell polarization are reverted by modulating HD early phenotypes. *Proc Natl Acad Sci U S A* **115**, E762-e771.

Dinamarca, M. C., Colombo, L., Tousiaki, N. E., Müller, M. and Pecho-Vrieseling, E. (2022). Synaptic and functional alterations in the development of mutant huntingtin expressing hiPSC-derived neurons. *Front Mol Biosci* **9**, 916019.

Galimberti, M., Nucera, M. R., Bocchi, V. D., Conforti, P., Vezzoli, E., Cereda, M., Maffezzini, C., Iennaco, R., Scolz, A., Falqui, A. et al. (2024). Huntington's disease cellular phenotypes are rescued non-cell autonomously by healthy cells in mosaic telencephalic organoids. *Nat Commun* **15**, 6534.

Haremaki, T., Metzger, J. J., Rito, T., Ozair, M. Z., Etoc, F. and Brivanlou, A. H. (2019). Self-organizing neuruloids model developmental aspects of Huntington's disease in the ectodermal compartment. *Nat Biotechnol* **37**, 1198-1208.

HD iPSC Consortium. (2017). Developmental alterations in Huntington's disease neural cells and pharmacological rescue in cells and mice. *Nat Neurosci* **20**, 648-660.

Laundos, T. L., Li, S., Cheang, E., De Santis, R., Piccolo, F. M. and Brivanlou, A. H. (2023). Huntingtin CAG-expansion mutation results in a dominant negative effect. *Front Cell Dev Biol* **11**, 1252521.

Lisowski, P., Lickfett, S., Rybak-Wolf, A., Menacho, C., Le, S., Pentimalli, T. M., Notopoulou, S., Dykstra, W., Oehler, D., López-Calcerrada, S. et al. (2024). Mutant huntingtin impairs neurodevelopment in human brain organoids through CHCHD2-mediated neurometabolic failure. *Nat Commun* **15**, 7027.

Liu, C., Fu, Z., Wu, S., Wang, X., Zhang, S., Chu, C., Hong, Y., Wu, W., Chen, S., Jiang, Y. et al. (2022). Mitochondrial HSF1 triggers mitochondrial dysfunction and neurodegeneration in Huntington's disease. *EMBO Mol Med* **14**, e15851.

Liu, Y., Chen, X., Ma, Y., Song, C., Ma, J., Chen, C., Su, J., Ma, L. and Saiyin, H. (2024). Endogenous mutant Huntingtin alters the corticogenesis via lowering Golgi recruiting ARF1 in cortical organoid. *Mol Psychiatry* **29**, 3024-3039.

Mattis, V. B., Tom, C., Akimov, S., Saeedian, J., Østergaard, M. E., Southwell, A. L., Doty, C. N., Ornelas, L., Sahabian, A., Lenaeus, L. et al. (2015). HD iPSC-derived neural progenitors accumulate in culture and are susceptible to BDNF withdrawal due to glutamate toxicity. *Hum Mol Genet* **24**, 3257-71.

Mehta, S. R., Tom, C. M., Wang, Y., Bresee, C., Rushton, D., Mathkar, P. P., Tang, J. and Mattis, V. B. (2018). Human Huntington's Disease iPSC-Derived Cortical Neurons Display Altered Transcriptomics, Morphology, and Maturation. *Cell Rep* **25**, 1081-1096.e6.

Monk, R., Lee, K., Jones, K. S. and Connor, B. (2021). Directly reprogrammed Huntington's disease neural precursor cells generate striatal neurons exhibiting aggregates and impaired neuronal maturation. *Stem Cells* **39**, 1410-1422.

Ring, K. L., An, M. C., Zhang, N., O'Brien, R. N., Ramos, E. M., Gao, F., Atwood, R., Bailus, B. J., Melov, S., Mooney, S. D. et al. (2015). Genomic Analysis Reveals Disruption of Striatal Neuronal Development and Therapeutic Targets in Human Huntington's Disease Neural Stem Cells. *Stem Cell Reports* **5**, 1023-1038.

Ruzo, A., Croft, G. F., Metzger, J. J., Galgoczi, S., Gerber, L. J., Pellegrini, C., Wang, H., Jr., Fenner, M., Tse, S., Marks, A. et al. (2018). Chromosomal instability during neurogenesis in Huntington's disease. *Development* **145**.

Disease Models & Mechanisms • Supplementary information

Smith-Geater, C., Hernandez, S. J., Lim, R. G., Adam, M., Wu, J., Stocksdale, J. T., Wassie, B. T., Gold, M. P., Wang, K. Q., Miramontes, R. et al. (2020). Aberrant Development Corrected in Adult-Onset Huntington's Disease iPSC-Derived Neuronal Cultures via WNT Signaling Modulation. *Stem Cell Reports* **14**, 406-419.

Świtońska, K., Szlachcic, W. J., Handschuh, L., Wojciechowski, P., Marczak, Ł., Stelmaszczuk, M., Figlerowicz, M. and Figiel, M. (2018). Identification of Altered Developmental Pathways in Human Juvenile HD iPSC With 71Q and 109Q Using Transcriptome Profiling. *Front Cell Neurosci* **12**, 528.

Wu, S., Hong, Y., Chu, C., Gan, Y., Li, X., Tao, M., Wang, D., Hu, H., Zheng, Z., Zhu, Q. et al. (2024). Construction of human 3D striato-nigral assembloids to recapitulate medium spiny neuronal projection defects in Huntington's disease. *Proc Natl Acad Sci U S A* **121**, e2316176121.

Xu, X., Tay, Y., Sim, B., Yoon, S. I., Huang, Y., Ooi, J., Utami, K. H., Ziaei, A., Ng, B., Radulescu, C. et al. (2017). Reversal of Phenotypic Abnormalities by CRISPR/Cas9-Mediated Gene Correction in Huntington Disease Patient-Derived Induced Pluripotent Stem Cells. *Stem Cell Reports* **8**, 619-633.

Zhang, J., Ooi, J., Utami, K. H., Langley, S. R., Aning, O. A., Park, D. S., Renner, M., Ma, S., Cheok, C. F., Knoblich, J. A. et al. (2019). Expanded huntingtin CAG repeats disrupt the balance between neural progenitor expansion and differentiation in human cerebral organoids. *bioRxiv*, 850586.

Disease Models & Mechanisms • Supplementary information
